# Measuring the Burden of Opioid-Related Mortality in Ontario, Canada, During the COVID-19 Pandemic

**DOI:** 10.1001/jamanetworkopen.2021.12865

**Published:** 2021-05-26

**Authors:** Tara Gomes, Sophie A. Kitchen, Regan Murray

**Affiliations:** 1 Keenan Research Centre of the Li Ka Shing Knowledge Institute, St Michael’s Hospital, Toronto, Ontario, Canada; 2 Leslie Dan Faculty of Pharmacy, University of Toronto, Toronto, Ontario, Canada; 3 Office of the Chief Coroner for Ontario, Toronto, Ontario, Canada

## Abstract

This cross-sectional study quantifies the added burden of fatal opioid overdoses during the first 6 months of the COVID-19 pandemic in Ontario, Canada.

## Introduction

The COVID-19 pandemic struck in the midst of an epidemic of opioid overdoses that has resulted in nearly 20 000 deaths in Canada since 2016.^1^ Pandemic-mandated measures, including changes to health care delivery to accommodate physical distancing and increased social isolation, can increase the risk of harm for people who use drugs.^2^ For example, reduced operation hours of health care services (eg, pharmacies and outpatient clinics) and harm reduction services (eg, drug checking programs and supervised consumption sites) have introduced additional barriers to care for people with opioid use disorder.^2^ Data from across North America suggest that the rate of opioid-related deaths has increased during the pandemic.^1,3,4^ We sought to quantify the added burden of fatal opioid overdoses occurring in Ontario during the first 6 months of the COVID-19 pandemic.

## Methods

This cross-sectional study was approved by the Unity Health Toronto research ethics board and followed the Strengthening the Reporting of Observational Studies in Epidemiology (STROBE) reporting guideline. We conducted a cross-sectional time series analysis of weekly fatal opioid overdoses among people aged 15 years or older in Ontario, Canada’s most populous province, from January 1, 2018, to September 20, 2020. We then compared fatal overdose rates and characteristics between three 6-month periods: the COVID-19 period (March 16 to September 15, 2020, beginning when a state of emergency was declared in Ontario); period 1, the same 6-month period in the prior year (March 16 to September 15, 2019); and period 2, the 6 months immediately before the COVID-19 period (September 14, 2019, to March 15, 2020). We obtained data on all confirmed or suspected opioid-related deaths from the Ontario Office of the Chief Coroner, including age; sex; rural or urban residence; whether fentanyl, stimulants, or benzodiazepines were detected; and whether naloxone was administered.^3^ An opioid-related death was defined as an acute intoxication or toxicity death resulting from the direct contribution of an opioid. Suspected opioid-related deaths were defined on the basis of evidence of drug use or paraphernalia found at the scene and/or an opioid detected in postmortem toxicology without final confirmation from the investigating coroner. We compared characteristics across the 3 periods using χ^2^ and *t* tests, with a type I error rate of .05 as the threshold for statistical significance. All tests were 2-tailed. Analyses were conducted in R version 4.0.4 (R Project for Statistical Computing). We used methods adapted from the Global Burden of Disease study^5^ to calculate the years of potential life lost (YLL) due to fatal opioid overdose.

## Results

Over the entire study period, the weekly number of opioid-related deaths increased 135%, from 23 to 54 deaths, with the most rapid growth occurring among those younger than 35 years (320% increase, from 5 to 21 opioid-related deaths weekly) (Figure). During the three 6-month periods of interest, a total of 2774 individuals (2037 [73.4%] men; 1311 [47.2%] aged 35-54 years) died from an opioid-related cause. In the first 6 months of the COVID-19 pandemic, 1237 people died of opioid-related causes (99.3 per million; 49 155 YLL) (Table). In contrast, 766 (62.4 per million; *P* < .001) and 771 (61.9 per million; *P* < .001) people died in period 1 and period 2, respectively, leading to 30 286 and 31 312 YLL, respectively. While most characteristics of fatal overdoses remained similar between periods, there were significant increases between period 2 and the COVID-19 period in the proportion of deaths among men (528 [68.5%] vs 948 [76.7%]; *P* < .001) and the prevalence of fentanyl (586 [76.0%] vs 1056 [85.4%]; *P* < .001) and stimulants (485 [62.9%] vs 880 [71.1%]; *P* < .001) (Table).

**Figure.**
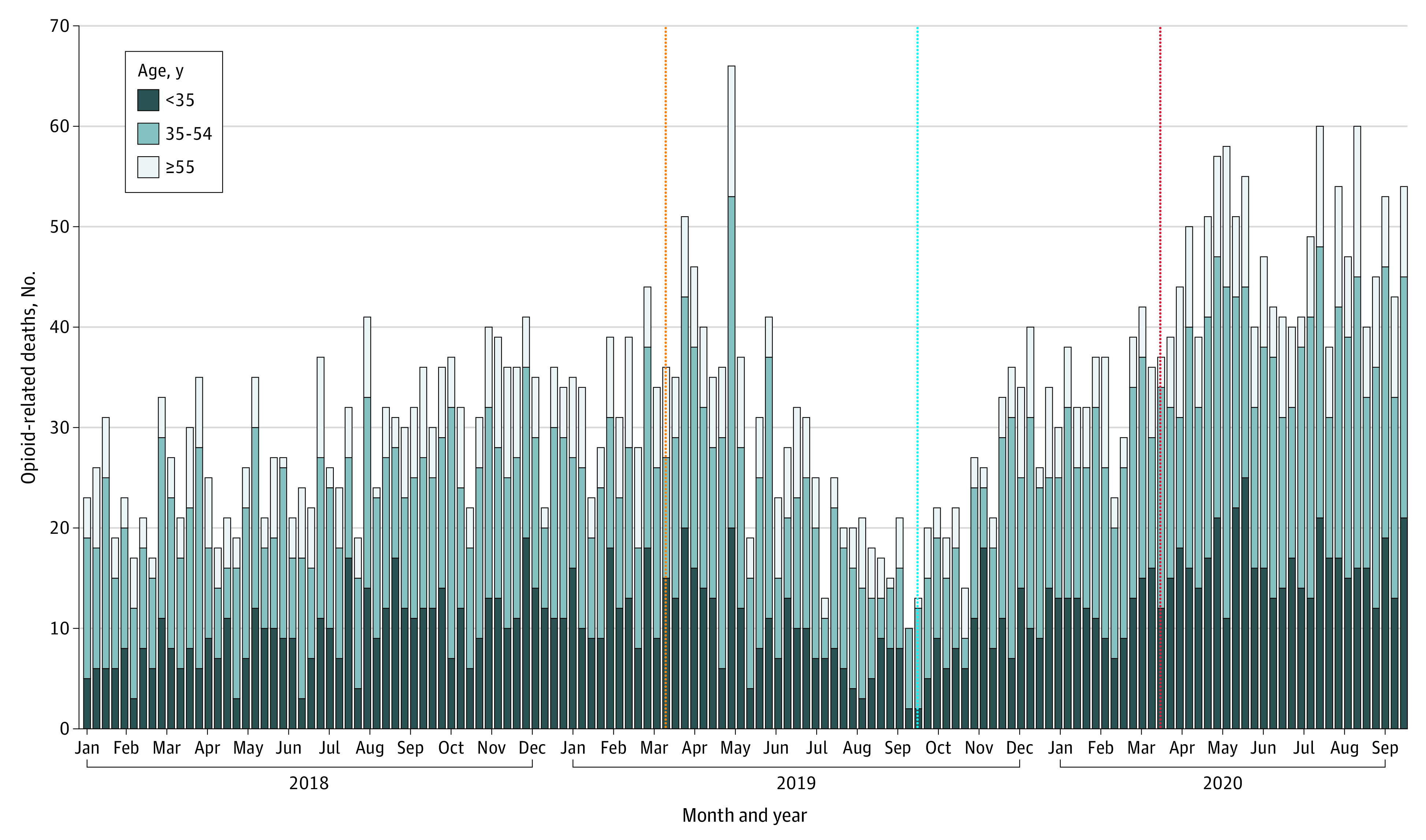
Time Series of Opioid-Related Deaths by Age Group, Ontario, Canada, January 2018 to September 2020 The vertical orange line indicates the start of period 1; vertical blue line, the start of period 2; vertical red line, the start of the COVID-19 period.

**Table.** Demographic Characteristics of Individuals Who Died From an Opioid-Related Cause During the First 6 Months of the COVID-19 Pandemic vs the Two 6-Month Periods Prior in Ontario, Canada

Characteristic	Decedents, No. (%)			*P* value	
	Period 1, March 16 to September 15, 2019	Period 2, September 14, 2019, to March 15, 2020	COVID-19 period, March 16, to September 15, 2020	Period 1 vs COVID-19 period	Period 2 vs COVID-19 period
Opioid-related deaths, No. (rate per million individuals)^a^					
Overall	766 (62.4)	771 (61.9)	1237 (99.3)	<.001	<.001
Individuals aged 15-34 y	249 (63.1)	274 (68.3)	426 (106.2)	<.001	<.001
Individuals aged 35-54 y	367 (96.3)	366 (95.9)	578 (151.5)	<.001	<.001
Individuals aged ≥55 y	150 (33.2)	131 (28.3)	233 (50.3)	<.001	<.001
Years of life lost (rate per 1000 individuals)	30 286 (2.5)	31 312 (2.5)	49 155 (3.9)	NA	NA
Sex					
Men	561 (73.2)	528 (68.5)	948 (76.7)	.08	<.001
Women	205 (26.8)	243 (31.5)	289 (23.3)		
Age, mean (SD)	41.8 (12.7)	40.8 (12.4)	41.5 (12.6)	.70	.11
Rurality^b^					
Rural areas	74 (9.7)	66 (8.6)	127 (10.5)	.66	.21
Small urban center	84 (11.0)	80 (10.4)	100 (8.3)	.03	.08
Medium urban center	115 (15.0)	122 (15.9)	194 (16.0)	.69	.93
Large urban center	478 (62.4)	487 (63.5)	778 (64.2)	.82	.90
Missing	15 (2.0)	12 (1.6)	13 (1.1)	.09	.32
Substances detected					
Fentanyl	587 (76.6)	586 (76.0)	1056 (85.4)	<.001	<.001
Stimulant^c^	515 (67.2)	485 (62.9)	880 (71.1)	.06	<.001
Benzodiazepine	205 (26.8)	273 (35.4)	450 (36.4)	<.001	.66
Naloxone administered	183 (23.9)	186 (24.1)	264 (21.3)	.18	.15

Abbreviation: NA, not applicable.

^a^ There were 0 suspected opioid-related deaths in period 1, 4 in period 2, and 25 in the COVID-19 period.

^b^ Data available among confirmed cases only.

^c^ Stimulants included cocaine, methamphetamine, and other stimulants.

## Discussion

Increasing rates of opioid-related deaths, particularly among young adults in Ontario, place a considerable burden on society. In the first 6 months of the COVID-19 pandemic, an additional 17 843 years of life were lost due to opioid overdose compared with the 6 months prior. A limitation of this study was that not all death investigations (29 of 2778 [1.0%] during the 3 periods) have concluded; however, it is likely that all of these will be confirmed as opioid related. The rising rates of harm among young adults as well as the increased contributions of fentanyl and stimulants to these deaths emphasize the urgent need for low-barrier access to evidence-based harm reduction services and treatment for opioid use disorder in all jurisdictions grappling with the overdose–COVID-19 syndemic.^6^
